# Agua4All: Providing Safe Drinking Water in Rural California Communities

**DOI:** 10.5888/pcd16.190165

**Published:** 2019-11-14

**Authors:** Anisha I. Patel, Amelie A. Hecht, Karla E. Hampton, Christina Hecht, Sarah Buck

**Affiliations:** 1 Division of General Pediatrics, Stanford University, Stanford, California; 2 Philip R. Lee Institute for Health Policy Studies, University of California, San Francisco, California; 3 Johns Hopkins Bloomberg School of Public Health, Baltimore, Maryland; 4 Intuitive Mind Consulting, Houston, Texas; 5 Nutrition Policy Institute at the University of California, Division of Agriculture and Natural Resources, Oakland, California; 6 RCAP Inc., Washington, District of Columbia; 7 Rural Community Assistance Corporation, Sacramento, California

## Abstract

**Introduction:**

Drinking water instead of sugar-sweetened beverages may reduce obesity and dental caries. Tap water is more affordable and sustainable than bottled water and more likely to contain fluoride, which prevents caries. To address inequities in access to safe tap water, cross-sector partners established the Agua4All safe drinking-water program in 2 rural San Joaquin Valley, California, communities. The program’s objective was to examine Agua4All’s feasibility, acceptability, and effect on water intake.

**Methods:**

We provided bottle-filling stations dispensing safe water at 12 sites in 2 communities and provided limited promotional support. To compare the effect of different levels of promotion, sites in 1 community also received a promotions toolkit, a stipend, and assistance in developing and conducting their own promotional activities (site-led promotion). Beverage intake at sites was observed at baseline (pre-installation), at time 1 (post-installation), and at times 2 and 3 (post-promotion). Flowmeters tracked water dispensings. Staff interviews examined implementation barriers and facilitators.

**Results:**

From baseline to time 3, a nonsignificant increase (21.16%) occurred in the proportion of people drinking water at sites with water stations and site-led promotion compared with sites with water stations and limited promotion (5.13%) (*P* = .14). Mean daily gallons of water taken from stations per site was 3.61 (standard deviation, 3.84). Most staff members (77%) at the sites preferred water stations to traditional drinking fountains.

**Conclusion:**

Bottle-filling stations with safe water and site-led promotion are a promising strategy for increasing water intake in communities without safe tap water. Larger studies should examine the effects of such stations on intake of sugar-sweetened beverages and on overall health.

**Summary**

**What is already known on this topic?**

Increasing access to safe and appealing drinking water in schools can increase intake of water and reduce consumption of sugar-sweetened beverages, and may help prevent obesity. No studies have investigated the effect of similar programs in communities that lack potable drinking water.

**What is added by this report?**

Community-wide installation of safe water bottle-filling stations, particularly when coupled with site-led promotion, may increase water intake in areas without safe drinking water.

**What are the implications for public health practice?**

As communities increasingly encounter contaminants in drinking water, the Agua4All program offers a short-term strategy for providing safe drinking water until longer-term infrastructure improvements are in place.

## Introduction

Drinking water instead of sugar-sweetened beverages (SSBs) can reduce calorie and sugar intake (1) and help prevent obesity and dental caries (2–5). A third of adults (6) and half of children are inadequately hydrated (7), and water is a healthy way to hydrate. Although either tap or bottled water can meet hydration needs, tap water is more affordable, leaves a smaller environmental footprint, and is more likely than bottled water to contain fluoride, which can prevent caries (8). Despite such benefits, low-income, minority populations drink less tap water than other groups, resulting in lower overall water intake, which has health implications (7,9,10). Low-income groups are also more likely to use limited financial resources to purchase bottled water (8,9).

Although low tap water intake is often attributed to the perception that bottled water is more convenient and better tasting (11), low consumption may also be due to concerns about tap water’s safety (12,13). In California’s San Joaquin Valley, the setting for this study, approximately 1.36 million residents who are predominantly low-income and Latino lacked access to safe drinking water in 2013 because of contaminants in the water, both naturally occurring and from agricultural and industrial activities (13).

Increased accessibility of appealing, safe tap water in schools has increased water intake (14,15,16,17), reduced SSB intake (15), and reduced obesity (16,17). Few studies have examined the effect of similar models in nonschool settings (18), and no study has investigated the effect of such programs in US communities with contaminated drinking water.

Our objective was to examine how bottle-filling stations dispensing safe water (hereinafter, water stations) coupled with either 1) limited promotion (signage, reusable water bottles provided by the study) or 2) limited promotion and the community’s own promotional activities (site-led promotion) affected intake of water in low-income, rural communities with a history of contaminated drinking water. Another objective was to understand community perceptions of the program and how it could be adapted for future use in other communities.

## Methods

### Study design and participants

Community-based participatory research enables communities to fully participate in the research process, from developing research questions to disseminating results (19). Researchers from the University of California, San Francisco, and the University of California Division of Agriculture and Natural Resources, advocates at Community Water Center, and the Central California Regional Obesity Prevention Program (a community-based organization) established an advisory board of representatives from schools, parks, water suppliers, community families, community-based organizations, and health departments. The focus of the partnership was to develop strategies to increase access to and intake of safe tap water among families in California’s San Joaquin Valley.

In 2014, 2 community partners received funding to establish Agua4All (http://www.rcac.org/agua4all), a program to ensure widespread access to safe, appealing drinking water in heterogeneous high-traffic locations throughout 2 communities in Kern County, an area of the San Joaquin Valley, including one community where the drinking water is contaminated with arsenic (20,21). The partners worked together to evaluate the Agua4All program.

Twelve sites in 2 Kern county communities, designated community A and community B, participated in the Agua4All study from November 2014 through June 2016. Community partners selected study sites on the basis of high community use, need, and public accessibility. Sites were health clinics, including those serving clients from the Special Supplemental Nutrition Program for Women, Infants, and Children (WIC), family resource centers, libraries, parks, and schools.

**Figure 1:**
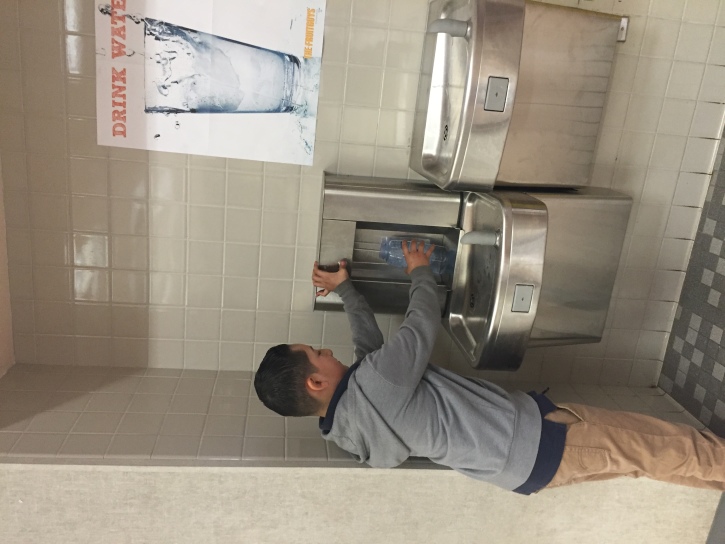
An Agua4All water station.

To compare different levels of drinking water promotion, the 6 community A sites received water stations (Figure 1), limited promotion, and support for site-led promotion to increase water intake; the 6 sites in neighboring community B received water stations and limited promotion but no site-led promotion support. Sites were matched on the basis of the location of the water station and the site type. For example, an outdoor station in a park in community A was matched to an outdoor station in a park in community B.

### Intervention activities

**Figure 2:**
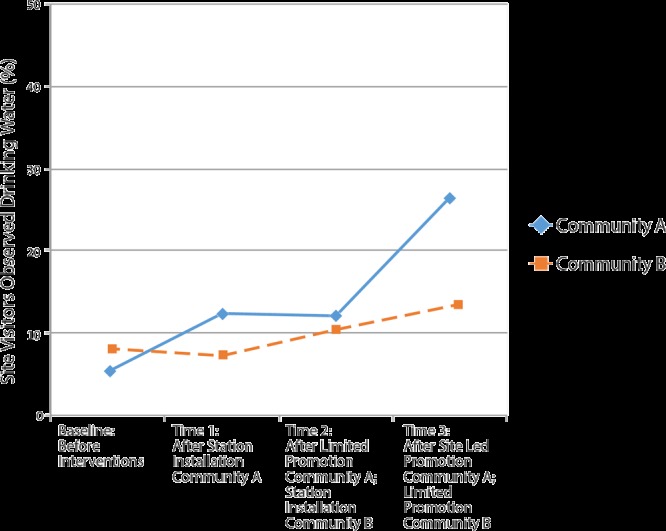
Percentage of visitors drinking water at 12 sites in San Joaquin Valley in the Agua4All Program, 2014–2016. A nonsignificant increase in consumption occurred among people drinking water at sites with water stations plus promotional activities that site staff developed and conducted compared with sites with water stations and limited promotional support (signage, reusable water bottles provided by study) (21.16% vs 5.13%, *P* = .14). Baseline data were collected from November 18, 2014 through December 16, 2014; time 1 data were collected from October 26, 2015 through January 22, 2016; time 2 data were collected from February 5, 2016 through April 23, 2016; time 3 data were collected from May 19, 2016 through June 15, 2016. Mean ambient temperature did not significantly differ between community A and community B at any time point.

Data were collected at baseline (pre-installation), time 1 (post-installation), and times 2 and 3 (post-promotion). Water station installation and related water promotion interventions were staggered. The stations were installed first in community A (August–October 2015) and then in community B (January–April 2016) (Figure 2). Sites installed at least 1 station capable of filling reusable water bottles; tap water was tested before installation. In community A, water at installation sites was found not to be contaminated, according to state and federal standards. In community B, drinking water violated the federal level for arsenic. To address this contamination, community partners installed filters certified by the California Department of Public Health to remove arsenic. Partners tested filtered water at initial installation, and filters were changed monthly. Stations were installed between baseline and time 1 in community A and between time 1 and time 2 in community B.

**Table:** 

Percentage of People Drinking Water at Study Sites	Community A	Community B
Baseline: before interventions	5.35	8.13
Time 1: After station installation in community A	12.41	7.27
Time 2: After limited promotion in community A ; station installation in community B	12.12	10.44
Time 3: After site-led promotion in community A; limited promotion in community B	26.51	13.26

Partners provided limited promotion at all sites, which consisted of 1) posting of multilingual signage promoting the safety and health benefits of water, 2) distribution of reusable water bottles (to the staff in all sites, students in schools), and 3) general program education. The safety of drinking water was communicated by disseminating drinking water test results through the program website, local news articles, and public relations events at sites. A program mascot, Wally the Water Droplet, was also displayed on all program materials and used to symbolize safe drinking water access. In schools, education also included an assembly and education curricula. Sites in community A received additional resources for site-led promotion including 1) evidence-based strategies to promote water consumption (22), 2) a stipend ($500 for schools, $250 for other sites), and 3) technical assistance from the community–academic research team to develop promotion. Site teams designed and implemented a range of promotion activities, which included the purchase of cups (at a WIC clinic and a library); development and posting of posters or banners about the health benefits of drinking water instead of packaged beverages and the importance of adequate hydration (at a WIC clinic, a library, and a park); distribution of stickers promoting the drinking of water (at health centers and libraries); purchase of additional reusable water bottles for the research staff to give away (at family resource centers, libraries, and schools), and announcements, assemblies, and contests related to water consumption (in schools). In community A, limited and site-led promotion was implemented after time 1; in community B, limited promotion was implemented after time 2 (Figure 2).

The primary outcome assessed was the proportion of people who used public drinking water sources at study sites. Community–academic researchers collected data at 4 time points during the study: 1) baseline (November–December 2014) before water interventions, 2) time 1 (October–November 2015) after installation of water stations in community A, 3) time 2 (February–April 2016) after limited and site-led promotion in community A and water station installation in community B, and 4) time 3 (May–June 2016) after limited and site-led promotion in community A and limited promotion in community B (Figure 2). During 3-hour observation periods, researchers documented the number of times people used all water sources (existing fountains or newly installed stations) and the number of people visiting the sites. Because of the large number of people visiting certain sites, researchers tallied each use of the water source rather than each individual using the source.

Researchers calculated the proportion of people using water sources by dividing the number of water source uses by the number of people at the site during each observation. In schools, researchers obtained this proportion by dividing the number of water source uses by the number of students in attendance on the observation day. At nonschool sites, observations occurred during times of peak visitor volume. In schools, observations occurred at lunch and recess. The 3-hour period was constant at each site (eg, the library was observed from 4:00 pm to 7:00 pm at each time point) to help control for unmeasured differences that could affect beverage intake and vary by time of day.

Researchers also tallied the number of people at sites who were in close proximity to the observed water sources with SSBs or bottled water and divided those counts by the total number of people visiting that area to obtain the proportions of visitors consuming either SSBs or bottled water.

Partners installed flowmeters on at least 1 new station per site to obtain the volume of water taken from stations post installation. Researchers calculated the gallons of water taken from the station per day by dividing the gallons taken between baseline and time 3 by the number of days the station was open to the public between flowmeter readings.

To explore barriers and facilitators to program implementation, researchers administered verbal surveys to 2 or fewer of the people staffing each site, including 9 site administrators, 3 staff members overseeing installation of the water station, and 1 staff member who served dual roles. In some cases, staff members worked at multiple sites. Open- and closed-ended questions assessed the respondent’s demographics and employee position; time spent on water station maintenance and upkeep, because this could increase program cost and adoption; attitudes about water stations; overall perceptions of water safety and quality, because such beliefs could influence use of new stations; and opinions about the overall program and suggestions for improvements. The study was approved by University of San Francisco’s Committee on Human Research.

### Data analysis

Researchers double-entered data by using the REDCap (REDCap Consortium) data entry system. To examine how installation of water stations and 2 approaches to promotion activities (limited promotion vs site-led promotion) affected water intake, we conducted mixed effects regressions, accounting for clustering of individuals in sites. Model 1 included the dependent variable (proportion of people at sites drinking water) and the independent variable (interaction of intervention status [stations vs no stations] and time [baseline vs time 1]), controlling for intervention status and time. Model 2 included the same dependent variable (proportion of people drinking water) and the independent variable (interaction of intervention status [stations/site-led promotion vs stations/limited promotion]) and time [baseline to time 3]), controlling for intervention status and time. Similar models examined how the interventions affected SSB and bottled water intake. Because ambient temperature may affect beverage intake, sensitivity analyses included ambient temperature in the models. One-way ANOVA examined variation in the magnitude of the intervention effect (baseline vs time 3) on the proportion of people drinking water by the type of site (eg, parks vs schools). Stata version 14 (StataCorp LLC) was used for analyses. *P* values of less than .05 were considered significant.

## Results

Communities participating in Agua4All were small (≤20,000 residents) and predominantly Latino, with approximately a third of their residents living below the federal poverty level (Table 1). At baseline, 9 sites had traditional fountains, 1 had no drinking water, and 2 had water dispensers that were only accessible to the staff (Table 2).

**Table 1:** Characteristics of San Joaquin Valley Communities (N = 12 study sites) Participating in the Agua4All Study, 2014–2016^a^

Characteristics	Community A, n = 6	Community B, n = 6
**Population, no.**	16,359	20,028
**Age of population, y, median**	24.8	24.2
**Male (%)**	51.2	53.7
**Race/ethnicity, %**		
Latino	95.3	91.3
White	3.5	6.9
Other	1.2	1.8
**Families with average annual income below federal poverty level, %**	34.6	29.0
**Educational attainment, %**		
<High school	63.2	65.5
High school	21.3	16.0
Some college	11.6	12.6
Associate’s degree or higher	3.9	5.9
**5th grade students overweight or obese, %**	57.1	52.3

a Based on US Census Bureau 2014 American Community Survey 5-year estimates (20).

**Table 2:** Daily Volume of Drinking Water Taken from Water Stations at Agua4All Study Sites (N = 12) in San Joaquin Valley Communities, 2014–2016^a^

Sites	Community A, Baseline Drinking Water Access	Community A, Water Use After Station Installation, Gallons	Community B, Baseline Drinking Water Access	Community B, Water Use After Station Installation, Gallons
Library	Lobby fountain	2.87	Lobby fountain	3.33
Clinic	Staff room water dispenser	1.79	Lobby fountain	1.33
Family resource center	No drinking water	0.95	Break room water dispenser	0.15
Community health center	Lobby fountain	9.75	Waiting room fountain	2.62
Park	Outdoor fountain	4.94	Outdoor fountain	12.76
School lunch	Cafeteria fountain	1.09	Cafeteria fountain	1.73
School recess^b^	Outdoor fountain (prekindergarten, kindergarten, general)	n/a	Outdoor fountain (prekindergarten, kindergarten, general)	1.75
All sites, gallons, mean^c^ (standard deviation)	Not applicable	3.56 (3.37)	Not applicable	3.65 (4.59)

a The daily volume of water taken from water stations was calculated by subtracting baseline flowmeter volume from the volume at the end of the study period. This volume was divided by the number of days the site was open to the public between flowmeter readings to obtain the daily volume of water used at each site.

b Not included in means for all sites because of lack of flowmeter readings from matched study site.

c Mean gallons of water used in study communities was not statistically different (*P* = .97).

In community A, the proportion of people drinking water increased following installation of stations (5.35% at baseline to 12.41% at time 1, difference 7.06%) but remained stable in community B where there were no changes in water access (8.13% at baseline to 7.27% at time 1; difference, −0.86%) with a difference in differences between communities of 7.92%, *P* = 0.11 (Figure 1). There was a greater increase in the proportion of people drinking water following station installation and site-led promotion (community A: 5.35% at baseline to 26.51% at time 3, difference 21.16%) compared with station installation and limited promotion (community B: 8.13% at baseline to 13.26% at time 3, difference 5.13%). Although the difference in these changes between communities was substantial, it was not significant (16.03%, *P* = .14). In sensitivity analyses controlling for ambient temperature, the increase in water intake following station installation was significant (difference in difference between communities from baseline to time 1 of 10.85%, *P* = .04). 

The largest increases in the proportion of people drinking water were in libraries, parks, clinics, and at school recess (15.61%–25.41%), and the smallest changes were observed in community health centers, family resource centers, and school cafeterias (0.72%–6.58%). Daily water taken from stations per site ranged from 0.15 to 12.76 gallons; the lowest estimates were in family resource centers, clinics, and schools and the highest were in community resource centers, libraries, and parks. There was no significant difference between communities in the amount of water (gallons) dispensed from stations (Table 2).

We saw no baseline differences in the proportion of people with SSBs or bottled water in sites between the study communities (SSBs: 0.01% community A vs 0.30% community B, *P* = .17; bottled water: 0.25% community A vs 0% community B, *P* = .34). There were no changes in the proportion of people drinking SSBs or bottled water from baseline to time 3 in community A versus community B following station installation and promotion (SSB: difference in difference 0.37%, *P* = .51; bottled water: difference in difference, 1.95%, *P* = .12).

Most (77%) site administrators and staff members overseeing station installations perceived drinking water as safe. They also reported a preference for stations over original water sources because of the newer age, functionality, and ease of access. Nearly all (95%) reported positive experiences participating in the program.

Improvements that respondents suggested for the program included creating maps of water stations and communicating about the safety of stations’ drinking water, the sugar content of various SSBs, and the health benefits of drinking water as an alternative to SSBs. Respondents also noted a need for funding to support ongoing promotion, continued upkeep of stations, water testing, filter changes, maintenance, and distribution of reusable water bottles to the community.

## Discussion

This quasi-experimental evaluation of a pilot community-wide program to provide access to and promotion of safe drinking water demonstrated promising trends in increasing water intake from public water sources. Although not significant, we saw an increase of nearly 8 percentage points in the proportion of people drinking water at sites with water stations compared with sites with traditional drinking fountains, and a 16 percentage point increase in the proportion of people drinking water at sites with site-led promotion compared with limited promotion. Although this pilot study was not powered to detect significant differences in beverage intake, results suggest that a combination of drinking water access improvements and targeted promotion are needed to substantially increase water intake. These findings should be explored in a larger study.

This study is one of the first to evaluate the effect of providing water access and promotion interventions in nonschool community settings (18). Previous evaluations of increased access to water bottle–filling stations in schools and in Philadelphia community recreation centers led to increases in water use from stations of approximately 10 to 15 gallons per day and increases in intake of 10% to 20%, which is within the range of effects observed in this study (14,15) (H. Lawman, personal communication, March 14, 2019).

In our study, we did not measure changes in SSB or bottled water intake. Although previous school-based studies suggest that water appeal and access interventions may prevent obesity (16,17), reductions in SSB intake have been observed only in 1 school-based study that evaluated how placing signage and cups next to traditional drinking fountains affected beverage intake (15). No studies have examined how such interventions affect intake of bottled water, which has environmental and cost implications. Future studies should examine how and what type of water promotion interventions in nonschool settings affect intake of SSBs and bottled water and associated health outcomes.

The amount of water taken from stations at different sites varied, ranging from 0.15 gallons at a family resource center to 12.76 gallons in a park. The greater use of stations in parks may relate to higher public traffic, a higher ambient temperature, and greater activity levels in those settings. Lastly, because park stations were easily accessible, residents may have filled containers with safe drinking water from park stations to consume at home. Given these findings, communities may want to consider policies to mandate the installation of water stations at newly constructed public sites that have more appeal and function than traditional fountains (23).

Although US law requires schools participating in the National School Lunch Program to provide water at no charge in cafeterias, simply providing water may not sufficiently increase water intake (24). In our study, station use in school settings was relatively low. A lack of cups near stations, rules that limited students from using stations after they were seated, offerings of other sweet beverages, and restricted access to stations in cafeterias beyond mealtimes all likely contributed to the low rates of water intake in school cafeterias. To maximize intake of water, schools may want to provide biodegradable or recyclable cups or supply reusable water bottles, remind students to get water during designated breaks, and increase access to stations throughout the school (25).

Qualitative data from administrators suggest that the program was feasible to implement and well received by the community. However, to promote intake of water instead of SSBs, it may be necessary to expand water stations to additional community locations, develop institutional policies to support intake of water instead of SSBs, and develop more robust water promotion strategies.

Although this study is one of the first to examine the effect of water access and promotion interventions in nonschool community settings, it had limitations. Because this was a quasi-experimental study, confounders (eg, site practices, policies related to nutrition) may not have been accounted for. Because this study was conducted in rural, agricultural communities with a history of contaminated tap water, findings may not be generalizable to dissimilar populations. Given the small sample size, our ability to detect significant differences in water and SSB intake was limited. Because of the close proximity of study communities, it is possible that people could have been exposed to site-led promotion occurring in the neighboring community. Lastly, because we did not collect data on the demographics of the people visiting study sites, we were unable to examine how these factors influenced consumption.

The Agua4All safe water station program, particularly when coupled with site-led promotion activities, appears to be a feasible and acceptable strategy to make safe drinking water available and to increase its intake in areas that lack access to safe drinking water. In 2017, California earmarked $9.5 million in funding to help schools provide safe drinking water (26). The state has contracted with several lead Agua4All partners to implement the grant program and to provide technical support to schools that need assistance. Although Agua4All provides a promising intermediate strategy for reducing unsafe drinking water access among vulnerable communities, advocacy is needed to ensure that ongoing resources are available so that safe tap water is available in the long term. With the 2019 enactment of the Safe and Affordable Drinking Water Fund (27) to improve access to safe drinking water in vulnerable California communities, evidence-based information on strategies to overcome challenges in providing drinking water should be used to inform decision-making.
